# Diseases of Wild Animals

**Published:** 1880-10

**Authors:** Jean Vilian


### ARTICLE V.

### *The Diseases of Wild Animals.*

BY PROFESSOR JEAN VILIAN.

Some naturalists have asserted that wild animals, when in a state ef liberty, are almost entirely free from disease, and that the latter afflicts them only when in captivity. I know that this is entirely erroneous, and it can be proved that captive wild animals are more exempt from ailments than those roaming at large.

While First Surgeon of the Thirty-first Regiment of the Line, then stationed at Alabera, in Algeria, I dissected the carcasses of about fifty lions. Tbe lungs of twenty of them were affected; one half of them were almost gone, showing that consumption is prevalent among the lions of the Sahara and the Sahel.

At the Jaadin des Plantes, here in Parie, seven lions have died since 1869. All of them were born here. I dissected them, and found that their lungs were entirely healthy. To what was the difference due? They received their food regularly, and were carefully protected from inclement weather, while the lions in Africa had to go without food for days, had to inhale the sandy air of the desert, and were frequently drenched by terrible rains.

There is at the Jardin des Plantes a wolf from the Ardennes. He was caught when about six years old. He was suffering from cough, and at one time we thought he was dying. He hawked and spat, and was always sullen and morose. Often he abstained from food for several days. At last we chloroformed him, and examined his throat.

He was found to be suffering from nasal catarrh in its most aggravated form. Under proper medical treatment he recovered rapidly. Nine wolves born at the Jardin never showed the slightest sign of disease.

M. Jacquemart, the famous Indian hunter, often told me that he had seen tigers spitting blood, which exhausted them so that they could be approached within a few feet with impunity.

All monkeys are very delicate animals. They are not gluttonous; and having so much exercise, they are rarely afflicted with diseases of the bowels. But they have weak lungs, and the reason why so many of the most interesting among them die when brought to Europe is the too sudden change of air, diet and water. There is no more intelligent monkey than the chimpanzee, a truly wonderful animal. While in Berlin I dined at the Zoological Gardens by the side of a pet chimpanzee. He partook of every dish like a human being, put sugar into his teacup, stirred it with the spoon, and drank the beverage with evident relish. But his eyes looked supernaturally bright. I felt his pulse. It was 125. "He will not live long," I said to his keeper.

"Why not?" he asked with a sorrowful mien.

"He is consumptive," I replied.

"Indeed? He often coughs."

The chimpanzee died a month later. His left lung was entirely gone.

Carnivorous animals suffer from digestive disorders only when fed upon poor meat. I dissected three hyenas; two in Paris, one in London. Their bowels presented an entirely healthy appearance, and so did their stomachs. But the reverse was the case of an old Abyssinian hyena belonging to a Greek menagerie-keeper, who had caught the animal himself in Africa. He managed to keep it alive for two years, but told me: "The beast always vomited, and often lay on the ground, moaning piteously. What was the cause?" I dissected the hyena. The stomach was in a terrible condition. It was dotted with the scars of boils.

Dogs are gluttons. Wild dogs are worse. We have at the Jardin one of these able to devour meat enough to gorge a tiger or a lion; but the animal has to pay dearly for its voracity; it is always suffering from aggravated constipation, and will not live long.

Foxes are shrewd about everything, and so they are about their food. What hunter has ever found a fox that died from disease? Zoologists admire the dissected body of a fox because there is never anything unhealthy to be found in its organs. Hence, foxes are long-lived.

Six months ago we received at the Jardin four buffaloes from the North American Plains. Two of them died three days after their arrival. They were found to be suffering from a multiplicity of diseases—dyspepsia, imperfect action of the kidneys, and fatty degeneration of the heart. The other two have been ailing ever since, and yet the young buffalo born at the Zoological Gardens of Cologne is the embodiment of health.

The elephant is one of the most temperate and abstemious of animals. He eats for his size so little food that it is a wonder how he is able to exist upon it. True, he dies in captivity before his time, but not from physical causes. There is no doubt that he is one of the most sensitive of animals. A slight or a disappointment mortifies him deeply. The elephants of South Africa, which are rough animals, when compared with those raised in captivity, die from diarrhœa or constipation, as Le Vaillant has stated. Their tamer brethren are free from disease; and, if they die before their time, they generally do so from the above-mentioned causes. Sultan, the pride of the Jardin, the most amiable elephant I ever knew, was unable to survive the death of his companion, the pet dog Jean.—*Popular Science Monthly.*

